# The Use of Artificial Intelligence in Creating Educational Material for Critical Care Clinicians: A Survey

**DOI:** 10.1111/nicc.70753

**Published:** 2026-10-28

**Authors:** Peter Nydahl, Susanne Krotsetis, Gero Langer, Lars Krüger

**Affiliations:** ^1^ Nursing Research University Hospital Schleswig‐Holstein Kiel Germany; ^2^ Institute of Nursing Science and Development Paracelsus Medical University Salzburg Austria; ^3^ Nursing Research University Hospital Schleswig‐Holstein Lübeck Germany; ^4^ Institute of Health, Midwifery and Nursing Sciences, Medical Faculty Martin Luther University Halle‐Wittenberg Halle (Saale) Germany; ^5^ Heart and Diabetes Center NRW, Care Directorate, Project and Knowledge Management/Care Development Intensive Care Ruhr University Bochum Bad Oeynhausen Germany

**Keywords:** artificial intelligence, education continuing, educational technology, healthcare professionals, surveys and questionnaires

## Abstract

**Background:**

One Minute Wonders (OMWs) are workplace‐based microlearning resources that support continuing professional development in healthcare. Generative artificial intelligence (AI) is increasingly used to create educational materials, but little is known about its application in OMWs development.

**Aims:**

To examine the use, experiences, opportunities and challenges related to AI‐assisted generation of OMWs as perceived among healthcare professionals participating in the German‐speaking OMW Network.

**Study Design:**

A cross‐sectional online survey was conducted among members of the German‐speaking OMW Network in Germany, Austria and Switzerland. A study‐specific questionnaire assessed AI use, quality assurance practices, perceived benefits and risks and educational support needs. Quantitative data were analysed descriptively, and free‐text responses underwent qualitative content analysis.

**Results:**

A total of 136 healthcare professionals were included, predominantly nurses (*n* = 128, 94.1%). Overall, *n* = 63 (46.3%) reported using AI to create OMW. Among AI users, AI was most used for generating figures (*n* = 41, 65.1%), graphics (*n* = 35, 55.6%) and design elements (*n* = 33, 52.4%), with ChatGPT being the most frequently used tool (*n* = 60, 95.2%). Most AI users critically reviewed AI‐generated content (*n* = 50, 79.4%). Inaccuracies requiring revision were reported by all AI users, most commonly ambiguous wording (*n* = 32, 50.8%), inappropriate images (*n* = 32, 50.8%) and incorrect content (*n* = 29, 46.0%). The content analysis identified a main theme for opportunities: AI as a facilitator of educational resource development and knowledge translation and a main theme for risks: AI as a potential threat to the trustworthiness and professional integrity of educational resources.

**Conclusions:**

The use of AI for developing educational materials such as OMWs seems to be established practice among participants of this study. AI offers benefits for efficiency and content creation but requires careful human oversight because inaccuracies and fabricated information are common.

**Relevance to Clinical Practice:**

AI can support the rapid development of workplace‐based educational resources. However, critical review and quality assurance are essential to ensure accuracy and safety.

## Impact Statements

- What is known about the topic
  ○ One Minute Wonders (OMWs) are educational materials for translating knowledge into practice, generated by nurses and other clinicians.
  ○ OMWs are one‐page resources that summarise evidence using brief texts and illustrations and can be read in 1 min.
  ○ OMWs can be placed on walls of units/wards, where waiting times appear, for example, beside blood gas analysers.
  ○ Within the German‐speaking OMW Network, thousands of OMWs have been created and shared.
- What this paper adds
  ○ Generative artificial Intelligence (AI) for generating OMWs is often used by nurses within the German‐speaking OMW Network
  ○ AI has several advantages in research, designing and drafting OMWs and reduces time‐consuming processes.
  ○ Around one third of all AI‐generated OMWs in this survey require revision due to mistakes such as false information, inappropriate figures, wrong references and others.
  ○ Prompts for AI‐generated OMWs are provided as illustrative examples to demonstrate the capabilities of AI and as practical templates to support readers in creating their own OMWs.

## 1 Introduction

Healthcare professionals working in acute and critical care settings are required to continuously update their knowledge and competencies to provide safe, effective and evidence‐based care [1]. However, increasing workloads and limited time for formal education challenge traditional approaches to continuing professional development [2, 3]. Consequently, workplace learning and microlearning have gained increasing attention in healthcare education [4, 5]. Microlearning refers to the delivery of focused educational content in small, manageable units that can be accessed within a short period of time and integrated into routine clinical work [6].

OMWs represent a practical application of this concept [7]. The latter are concise one‐page educational resources that summarise key knowledge, clinical procedures, or evidence‐based recommendations in a visually accessible format. In clinical practice, OMWs are typically developed by qualified nurses who identify a practice‐relevant question, conduct a literature search, select and critically read a relevant scientific paper and summarise its key findings with supporting illustrations and the full reference. Where possible, an expert reviews the OMWs for accuracy and quality before dissemination; the entire process usually takes approximately 1–2 h [8]. They are often used in intensive care units (ICUs) and displayed in areas where healthcare professionals experience brief waiting periods during their workday, such as near blood gas analysers, thereby transforming otherwise unused time into learning opportunities. The OMW concept originated in clinical practice and has spread widely across German‐speaking countries and beyond. Rowlinson [7] first described OMWs in the UK as a workplace‐based educational strategy designed to support learning during routine clinical activities. Furthermore, an OMW Network for English‐speaking countries was founded recently [7]. More recently, Krüger, Mannebach [9] reported widespread implementation of OMWs across various healthcare settings and positive perceptions among users regarding their educational value and practicality. OMWs align with principles of workplace learning, just‐in‐time learning and cognitive load theory by providing concise and focused information that can be accessed directly within the clinical environment. Nevertheless, despite their growing popularity, scientific evidence and best practice regarding their development, implementation and use remains limited. Since 2018, a German‐speaking OMW Network has grown to approximately 900 members, supporting the development and dissemination of OMWs through newsletters, hospital websites and scientific journals [8], Krüger, Mannebach [9], [10, 11]. Moreover, OMWs have also been used in the context of research projects [12, 13].

At the same time, generative artificial intelligence (AI), referring to AI systems that can generate new content such as text, images, or other media in response to user prompts, is increasingly being integrated into healthcare education [14, 15, 16]. Recent reviews suggest that AI can support the rapid creation of educational materials, facilitate knowledge translation and reduce the workload associated with content development [17]. OMWs are a relevant use case for generative AI because their development involves summarising complex information, presenting it in an understandable way and combining text with visual elements. However, their highly condensed format requires careful human review to ensure that the underlying evidence remains accurate and is not misinterpreted. Healthcare professionals can now use AI tools to generate text, summaries, illustrations, visual abstracts and educational resources within seconds, which may facilitate generation when appropriate prompts are used [18]. Prompt engineering refers to the systematic development and refinement of instructions that guide AI in generating OMWs with the intended content, structure and format. More information is reported in the Box 1.

BOX Infobox: Prompt Engineering. 1 Independently of the present survey, the authors explored the technical feasibility of generating OMWs from scientific publications using generative AI and iteratively developed structured prompts for this purpose. Examples are provided in this publication solely to illustrate this potential and were neither used nor evaluated by survey participants. The prompts used to generate the sample OMWs are provided in the Supplement to enhance transparency and reproducibility and to enable readers to test, critically evaluate, adapt and further develop the approach. They are intended as illustrative starting points rather than a validated formula. Adding prompts to a manuscript ensures reliability in science. The prompt specified visual design principles, content hierarchy, scientific reporting standards, ethical considerations and disclosure requirements. The complete prompt is provided in the supplement, with three examples (33–35) illustrating how a large language model can transform a scientific publication into a concise workplace‐learning resource following predefined design and content rules.

Through this approach, generative AI can support different educational paradigms, including evidence translation, narrative learning and values‐based professional development. Although the opportunities and challenges of AI in health professions education have been widely discussed, little is known about its application in workplace‐based microlearning formats. In particular, it remains unclear to what extent healthcare professionals use artificial intelligence to create OMW.

## 2 Background

One Minute Wonder (OMW) is workplace‐based microlearning materials that provide concise educational content for healthcare professionals during routine clinical activities. At the same time, generative artificial intelligence (AI) is increasingly being used to create educational materials. However, little is known about the extent to which healthcare professionals use AI to develop OMWs.

## 3 Aims

To examine the use, experiences, opportunities and challenges related to AI‐assisted generation of OMWs as perceived among healthcare professionals participating in the German‐speaking OMW Network.

## 4 Method

A cross‐sectional online survey was conducted to explore the use of AI for the creation of OMWs among healthcare professionals. The study was designed and reported in accordance with the Consensus‐Based Checklist for Reporting of Survey Studies (CROSS) [19].

### 4.1 Setting and Participants

The survey was distributed to all German‐speaking OMW Network members, comprising healthcare professionals from Germany, Austria and Switzerland who use or are interested in OMWs as a workplace‐based educational strategy [9]. All registered members of the network were eligible to participate and were invited by email. Participation was voluntary and no incentives were offered.

### 4.2 Survey Instrument

A study‐specific questionnaire was based on a previous survey in this network and developed in German by the research team to investigate the use of AI in the development of OMWs [9]. The questionnaire comprised 27 items organised into five domains: (1) socio‐demographic characteristics, (2) use of AI for OMWs creation, (3) handling of scientific sources and copyright‐related issues, (4) quality assurance and error detection and (5) perceptions, future perspectives and support needs. The survey included dichotomous questions, multiple‐choice questions, Likert‐type scales, numerical entries and open‐ended questions.

The questionnaire used predefined conditional branching according to participants' reported use of AI for OMWs generation. Participants reporting previous AI use were directed to questions addressing their experiences with AI‐assisted OMWs generation, whereas participants without such experience skipped these questions and proceeded to open‐ended questions on perceived opportunities, risks and support needs related to AI use. Thus, experience‐specific questions were analysed only for participants to whom they were applicable. Participants with AI use are reported in an analysis of experience‐specific questions in this survey.

The full questionnaire is provided as Supporting Information. Prior to administration, the questionnaire was pilot tested for relevance, comprehensiveness and clarity with seven healthcare professionals, representing the target population and experience in research methods. Based on participant feedback, minor revisions to wording and question clarity were made. No substantial changes to the questionnaire structure or content were required. As the questionnaire was designed to describe practices and experiences rather than to measure a latent construct using a multi‐item scale, psychometric testing of internal consistency was not considered applicable.

### 4.3 Survey Administration

The survey was administered electronically using SurveyMonkey (Momentive Inc., San Mateo, CA, USA). Data collection took place between 8 and 21 June 2026. An initial invitation email containing information about the study and a survey link was distributed to all German‐speaking OMW Network members. One reminder email was sent during the recruitment period after 7 days. Participation was anonymous and respondents could complete the questionnaire using their own electronic devices. SurveyMonkey settings were configured to minimise duplicate participation where technically feasible.

### 4.4 Data Analysis

Quantitative data were exported from SurveyMonkey and analysed using JASP (Version 0.19.3, University of Amsterdam, Amsterdam, The Netherlands). Categorical variables were summarised using frequencies and percentages. Continuous variables were described using appropriate measures of central tendency and dispersion. Associations between categorical variables were analysed using Fisher's exact test. A two‐sided *p* value < 0.05 was considered statistically significant. No additional assumptions regarding data distribution were required for this analysis.

Responses to the open‐ended questions were provided and analysed in German language using qualitative content analysis according to Mayring [20]. An inductive approach was used, with codes and themes developed from the participants' responses rather than defined a priori. Qualitative content analysis was used for the open‐ended questions because responses were generally brief and the aim was to systematically condense the data, identify recurring meanings and provide an overview of participants' perspectives. Given the brief nature of the open‐ended responses, AI‐assisted qualitative analysis was used only to suggest initial coding and theme development using ChatGPT with GPT‐5.5 (OpenAI, USA). Two researchers independently reviewed the suggested codes and themes against the original responses, revised them where necessary and approved the final analysis. Reflexivity was supported by comparing codes and themes with the original responses, discussing ambiguous interpretations and considering the potential influence of the researchers' experience with OMW. Inter‐rater reliability was not quantified, as the purpose of researcher comparison was to support interpretative refinement and consensus rather than to assess statistical agreement between independent coders. The themes and illustrative quotations reported in this manuscript were subsequently translated into English using ChatGPT using GPT‐5.5 (OpenAI, USA), with the final translations reviewed and approved by the authors.

Open‐ended responses were analysed across the overall sample, as the qualitative analysis aimed to identify overarching perspectives rather than differences according to previous AI experience.

Missing responses were not imputed. Analyses were conducted on an available‐case basis.

### 4.5 Ethical Considerations

Participation was voluntary and anonymous. Before accessing the questionnaire, participants received written information regarding the study objectives, data handling procedures and the intended publication of aggregated findings. Completion of the survey was considered informed consent to participate.

No personally identifiable information was collected, stored, or analysed. According to applicable institutional and national regulations, formal ethics committee approval was not required because the study involved an anonymous survey of healthcare professionals and did not collect sensitive personal data. The study was conducted in accordance with the ethical principles of the Declaration of Helsinki [21].

## 5 Results

Out of 1038 invited network members, *n* = 147 (14.2%) participated in the survey. Excluded from analysis were *n* = 11 (7.4%) with *n* = 2 (1.4%) gave no consent and *n* = 9 (6.1%) providing no answers, leaving *n* = 136 (92.5%) for analysis.

The majority of respondents were nurses (*n* = 128, 94.1%), had an academic degree (*n* = 93, 68.4%) and worked in the ICU (*n* = 48, 35.3%) (Table 1).

**TABLE 1:** Respondents' Characteristics.

Item	*n* (%)
Profession	
Nurses	128 (94.1)
Physicians	1 (0.7)
Others (not specified)	7 (5.1)
Highest education	
Academic degree	93 (68.4)
Vocational degree	43 (31.6)
Place of work	
Intensive Care Unit	48 (35.3)
General Ward	20 (14.7)
Education/Nursing School	16 (11.8)
Management	12 (8.8)
Emergency Department	6 (4.4)
Specialist Clinical Department	1 (0.7)
Other (not specified)	33 (24.2)

In total, *n* = 63 (46.3%) participants reported using AI for generating OMW, whereas *n* = 73 (53.7%) had not yet used AI for this purpose. There was no statistically significant difference in AI use between participants with and without an academic degree (*n* = 48 (76.2%) vs. *n* = 15 (23.8%), *p* = 0.069).

### 5.1 Planned Analysis of Experience‐Specific Questions Among Participants Using AI

Following results refer to participants who used AI in generating OMWs (*n* = 63). These participants judged their self‐perceived competency in using AI for generating OMWs on a scale from 1 to 4 (4 = highest) in median as two (Interquartile Range (IQR) 2–3).

Participants reported generating 133 OMWs, with a median 2 (IQR 2–4) and a maximum of *n* = 30 per participant. Most of the AI‐using participants reported using AI for generating figures (*n* = 41, 65.1%), graphics (*n* = 35, 55.6%), or design (*n* = 33, 52.4%) and used most often ChatGPT (*n* = 60, 95.2%), Microsoft Copilot, or Gemini (*n* = 11, 17.5% each). Only a minority used a paid subscription version vs. a free version (Table 2).

**TABLE 2:** Use of Artificial Intelligence for generating One Minute Wonder.

Item	*n* (%)
Purpose of using AI^a^	
Figures	41 (65.1)
Graphics, pictograms	35 (55.6)
Design	33 (52.4)
Generating text	27 (42.9)
Improving language	25 (39.7)
Summaries	24 (38.1)
Literature research	14 (22.2)
Translations	9 (14.3)
Other^b^	6 (9.5)
Type of AI^a^	
ChatGPT	60 (95.2)
Microsoft Copilot	11 (17.5)
Gemini	11 (17.5)
Claude	9 (14.3)
Canva AI	7 (11.1)
DALL E	3 (4.8)
Others^c^	12 (19.0)
Version	
Free version	37 (58.7)
Paid subscription version	16 (25.4)

Abbreviation: AI, Artificial Intelligence.

^a^ Multiple answers, percentages might not round up to 100% due to rounding.

^b^ Pictogram, sketch notes, research, reducing word count, corrections, brainstorming.

^c^ Perplexity, Notebook LM, hospitals' own AI.

Uploading content was common and most AI‐using participants reported uploading their own texts (*n* = 33, 52.4%), scientific articles (*n* = 30, 47.6%), clinical guidelines (*n* = 25, 39.7%) and others (Table 3).

**TABLE 3:** Uploads to Artificial Intelligence for generating One Minute Wonder.

Item	*n* (%)
My own texts	33 (52.4)
Scientific articles	30 (47.6)
Clinical guidelines	25 (39.7)
Images/graphics	21 (33.3)
Abstracts	14 (22.2)
Presentations	7 (11.1)
Anonymised case data	5 (7.9)
Other: copy & paste	1 (1.6)
No uploads	11 (17.5)

The majority of AI‐using participants reported use of AI for generating OMWs occasionally to regularly (*n* = 17, 27.4% each), uploading materials only rarely (*n* = 34, 54.8%), almost always considering data protection (*n* = 37, 63.8%) and critically reviewing AI‐generated content (*n* = 50, 79.4%) (Table 4).

**TABLE 4:** Uploads of Content and Errors in Using Artificial Intelligence for Generating One Minute Wonder.

How often do you …	Frequency^a^				
	Rarely	Occasionally	Regularly	Frequently	Almost always
Use AI for generating OMW	19 (30.6)	17 (27.4)	17 (27.4)	3 (4.8)	6 (9.7)
Perform uploads on AI	34 (54.8)	14 (22.6)	7 (11.3)	4 (6.4)	3 (4.8)
Consider data protection or copyright issues when using AI	8 (13.8)	3 (5.1)	4 (6.9)	6 (10.3)	37 (63.8)
Critically review AI‐generated content in OMWs that you create yourself	0 (0)	2 (3.1)	4 (6.3)	7 (11.1)	50 (79.4)
Identify factual errors or inaccuracies during the AI‐assisted creation of OMWs	8 (12.7)	19 (30.2)	22 (34.9)	9 (14.3)	5 (7.9)
Revise AI‐generated OMWs	1 (1.6)	12 (19.7)	26 (42.6)	9 (14.7)	13 (21.3)

Abbreviations: AI, Artificial Intelligence; OMWs, One Minute Wonders.

^a^ *n* (%), Frequency were defined as: Rarely = approximately 0–1 out of 10 OMW; Occasionally = approximately 2–3 out of 10 OMW; Regularly = approximately 4–6 out of 10 OMW; Frequently = approximately 7–8 out of 10 OMW; Almost always = approximately 9–10 out of 10 OMW.

All AI‐using participants reported errors and inaccuracies during the AI‐based production process, requiring revisions of the OMW. Errors and revisions were reported occasionally to regularly (Table 4), especially in ambiguous wording and inappropriate images (each *n* = 32, 50.8%) (Table 5).

**TABLE 5:** Errors and inaccuracies in Artificial Intelligence assisted generation of One Minute Wonder.

Item (multiple answers)	*n* (%)
Ambiguous wording	32 (50.8)
Inappropriate images	32 (50.8)
Incorrect content	29 (46.0)
Fabricated references	28 (44.4)
Oversimplification	17 (27)
Outdated information	15 (23.8)
Incorrect numerical data	12 (19.0)
No problems identified	3 (4.8)
Other (please specify)^a^	7 (11.1)

Abbreviation: AI, Artificial Intelligence.

^a^ Others were in alphabetical order: Erroneous or misleading text outputs; Formatting and display problems; Inaccuracies requiring prompt revision; Incorrect translations; Image generation errors (e.g., distorted or incorrect text); Spelling and language errors.

Most of the AI‐using participants agreed that AI‐generated OMWs should be labelled as such (*n* = 47, 74.6%), as well as *n* = 13 (20.6%) who were unsure, and *n* = 3 (4.8%) disagreed.

### 5.2 General Opportunities and Risks

All AI‐using and non‐using participants reported several themes concerning the opportunities and risks associated with using AI in generating OMW.

#### 5.2.1 Opportunities

Of the 136 participants, *n* = 111 (81.6%) provided a free‐text response to the question regarding perceived opportunities of AI in the creation of OMW.

The qualitative content analysis identified one main theme: *AI as a facilitator of educational resource development and knowledge translation*. Participants perceived AI as a multifaceted tool that supports the efficient development, adaptation, visualisation and dissemination of educational content. Beyond reducing workload, AI was described as facilitating access to evidence, enhancing communication and design and stimulating creativity throughout the OMWs development process.

Illustrative quotations included:

P16: AI can provide significant support – especially in making OMWs more visual or formulating good memorable phrases. The OMWs are easier to remember and are more likely to be read because they look more appealing.

P21: Faster, with more attractive visual/graphic design. People have a very short attention span nowadays and often will not even read a single A4 page of text unless there is something that catches their eye, for example a comic‐style illustration.

Five subthemes were identified: *efficiency, visualisation, communication, knowledge and creativity*. Participants most frequently described *efficiency*, highlighting faster content production, text summarisation and reduced workload during OMWs creation. *Visualisation* was another dominant theme, with respondents emphasising AI‐generated images, graphics and attractive layouts that could increase attention and improve learning. Under *communication*, participants valued assistance with text formulation, simplification, target‐group adaptation and translation. *Knowledge* reflected perceived benefits in accessing scientific information, identifying relevant literature and obtaining up‐to‐date knowledge. Finally, *creativity* captured the role of AI in providing ‘new ideas’, ‘design ideas’ and ‘additional perspectives’ for the development and implementation of OMWs (Table S1).

#### 5.2.2 Risks

The same number of participants provided responses to the question regarding risks and problems. The qualitative content analysis identified a main theme: *AI as a potential threat to the trustworthiness and professional integrity of educational resources*. Participants expressed concerns that AI‐generated OMWs may compromise the accuracy, scientific rigour, authenticity and ethical integrity of educational materials. Across themes, respondents emphasised the need for human oversight, critical appraisal and transparency to ensure that AI supports rather than replaces professional judgement and engagement.

Example quotes are:

P9: Reliable use of trustworthy scientific sources varies greatly depending on the AI. AI‐generated content basically always needs to be checked again for accuracy and, if necessary, revised. This is also a major time‐consuming factor.

P28: I am very cautious about OMWs generated entirely by AI, because you can never be completely sure where the information comes from. Current AI chatbots are still not good enough to rely on the information with even 85% confidence.

Subthemes were *misinformation, need for verification, scientific quality, loss of professional engagement, legal and ethical concerns and loss of authenticity*. The dominant concern was *misinformation*, with participants frequently referring to false information, hallucinations, inaccurate content and fabricated references. Closely linked was the *need for verification*, as respondents repeatedly emphasised that AI‐generated outputs require careful professional review and validation. Concerns regarding *scientific quality* focused on unreliable sources, missing evidence and inadequate literature appraisal. Participants also described a potential *loss of professional engagement*, including reduced critical thinking and less intensive engagement with content. *Legal and ethical concerns* encompassed data protection, copyright and transparency issues. Finally, respondents highlighted a potential *loss of authenticity*, including reduced individuality, generic outputs and diminished contextual relevance (Table S2).

### 5.3 Educational Needs

Participants reported multiple education needs related to generating OMWs by using AI, such as training courses (*n* = 81, 59.6%), quality assurance checklists (*n* = 79, 58.1%), example prompts (*n* = 73, 53.7%) and other (Table 6). Comparing results for participants with vs. without experience in using AI in generating OMWs led not to statistically significant results (all *p* > 0.05).

**TABLE 6:** Educational needs by clinicians with vs. without experience in using Artificial Intelligence for generating OMW.

Item (multiple answers)	Total^a^ (*n* = 136)	With AI experience^a^ (*n* = 63)	Without AI experience^a^ (*n* = 73)	*p* ^b^
Training courses	81 (59.6)	40 (63.5)	40 (55.6)	0.349
Quality assurance checklists	79 (58.1)	35 (55.6)	43 (59.7)	0.625
Example prompts	73 (53.7)	37 (58.7)	35 (48.6)	0.240
Exchange within the OMW network	71 (52.2)	28 (44.4)	42 (58.3)	0.107
Image databases	63 (46.3)	25 (39.7)	37 (51.4)	0.173
Data protection recommendations	49 (36.0)	22 (34.9)	26 (36.1)	0.885
Guidelines	45 (33.1)	20 (31.7)	24 (33.3)	0.844
No support needed	10 (7.4)	3 (4.8)	6 (8.3)	0.407
Other^c^	1 (0.7)	1 (1.6)	0 (0)	n/a

Abbreviations: AI, Artificial Intelligence; n/a, not applicable; OMW, One Minute Wonder.

^a^ Data reported as *n* (%).

^b^ Fishers Exact Test.

^c^ Other: stringent labelling of AI‐generated content.

### 5.4 Potential Use and Concerns

The majority of participants (*n* = 99, 73.3%) reported that they would use AI‐generated OMWs in practice, with a significant difference between participants using AI vs. non‐using (*n* = 53 (84.1%) vs. *n* = 46 (63.9%), *p* = 0.025). Concerns were expressed by *n* = 50 (79.4%) of AI users vs. *n* = 60 (82.2%) of non‐AI users, *p* = 0.827. In total, *n* = 29 (21.3%) encountered the dissemination of incorrect AI‐generated content, *n* = 49 (36.0%) had not, and *n* = 43 (31.6%) were unsure. Participants who reported experiences with AI‐related problems most frequently described fabricated or inaccurate references, incorrect factual information and errors in AI‐generated images, also outside the context of OMW. Several respondents noted examples of non‐existent studies, references that could not be verified and outdated evidence, particularly in wound care. Other reported issues included incorrect numerical information, inappropriate clinical illustrations and erroneous clinical statements, such as using a 24‐h ECG for blood pressure measurement.

To illustrate the application of AI‐generated OMW, the authors created an OMW using prompt 1 (supplement). The OMWs was critically reviewed and revised by the authors before final approval and was not part of the survey or generated by participants (Figure 1).

**FIGURE 1:**
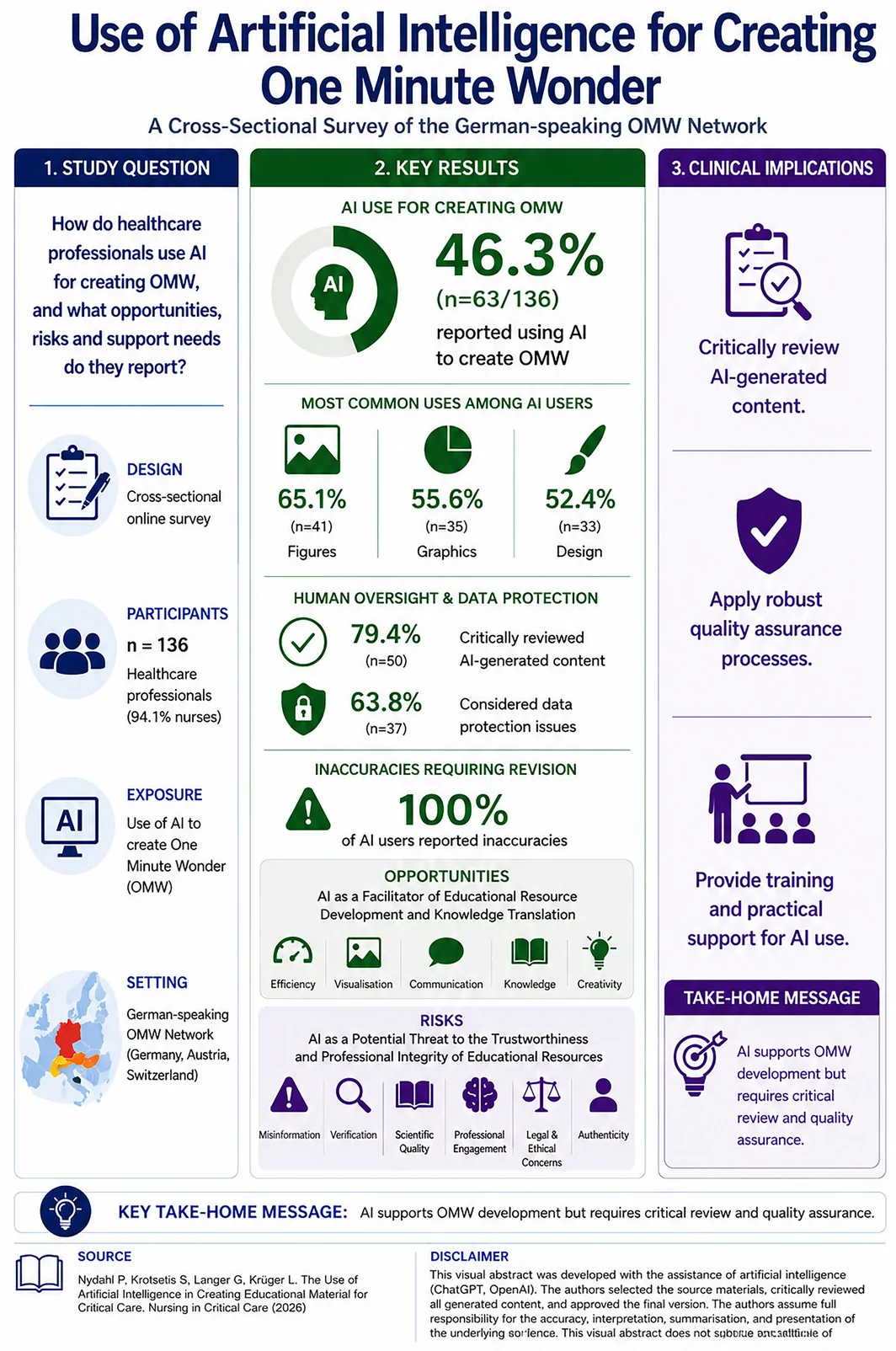
One Minute Wonder, summarising the main results of this study. Visual summary of the cross‐sectional survey conducted within the German‐speaking OMW Network. The figure presents the study design and sample, the proportion of respondents using artificial intelligence (AI) to create OMWs, common applications of AI, human oversight and data protection considerations, reported inaccuracies requiring revision, perceived opportunities and risks and implications for clinical practice. Percentages relating to AI use are based on respondents who reported using AI for OMW creation unless otherwise indicated. AI, artificial intelligence; OMW, One Minute Wonder.

## 6 Discussion

In this survey with nearly 140 nurses and other clinicians within the German‐speaking OMW Network, half of participants reported experience of using AI to generate OMWs for educational purposes. Most AI‐using participants reported using freely available AI tools, especially for generating figures, graphics, or design. The majority considered data protection and critically reviewed AI‐generated content. All AI‐using participants reported errors and inaccuracies during the AI‐based production process, requiring revisions of the OMW. AI was perceived as both a facilitator of educational resource development and knowledge translation and a potential threat to the trustworthiness and professional integrity of educational resources. Participants suggested that AI‐generated content should be labelled as such, and expressed a need for further training, quality assurance checklists, example prompts and other support.

The AI tools used are unsurprising and consistent with those mentioned in research reports [22, 23]. In addition to generating images, AI is also used to generate text for OMW. Tseng, Huang [22] reported a better academic report writing among nursing students using ChatGPT and Copilot, compared to traditional teaching methods. When creating OMW, concise summaries are important so that the content can be absorbed quickly in 1 min, in keeping with the principles of microlearning [4]. AI may therefore support the development of concise text for OMWs [24]. On the other hand, nearly all AI‐using participants reported errors and inaccuracies in initial outputs. Authors of OMWs therefore need sufficient domain expertise to critically verify generated content against current evidence before dissemination.

The growing use of generative AI for producing OMWs and other educational resources requires robust governance frameworks beyond technical innovation. First, generative AI may reproduce or amplify existing biases, including racial, cultural, gender and socio‐economic stereotypes embedded in training data, potentially resulting in inequitable educational messages or visual representations [25]. Consequently, AI‐generated OMWs should undergo expert review to ensure fairness, inclusivity and alignment with professional values [8, 26]. Moreover, large language models may generate inaccurate, misleading, or entirely fabricated health information (‘hallucinations’), including fictitious diseases, incorrect references, or outdated recommendations [27]. These considerations are particularly relevant when educational materials include patient images, case reports, wounds, delirium, or vulnerable populations such as children, people with disabilities, neurodivergent individuals, or ethnic minorities, placing specific responsibilities and competencies on authors of OMWs in nursing care [28]. Defining the competencies needed for responsible AI‐assisted educational resource development and integrating these into healthcare professional education [29] represents important research priorities [30]. AI‐assisted generation of educational materials must comply with emerging legal and ethical frameworks, including the European Union Artificial Intelligence Act, which emphasises transparency, human oversight, accountability, data protection, non‐discrimination, intellectual property and the protection of fundamental rights [31]. Further research is needed to explore how users recognise and address concerns related to AI‐generated OMWs and which strategies they apply to mitigate potential risks during their development.

Standardised quality assurance processes should accompany AI‐assisted generation of educational resources. Future quality checklists should include mandatory disclosure of AI use, authorship and accountability statements, evidence verification, copyright compliance, intended educational use, version control and digital trust markers such as QR codes linking to methodological information, source publications, prompt documentation and quality assurance criteria [25, 32]. Based on the findings of this study and considerations specific to generative AI, we propose the Quality Checklist for AI‐Assisted One‐Minute Wonder (Q‐AI‐OMW) as a preliminary framework for developing and reviewing AI‐assisted OMWs [33]. It addresses five quality domains: educational quality, visual and information design, clinical and scientific quality, AI‐specific quality assurance and responsible and transparent use (supplement). The checklist requires further evaluation and validation before its use as a standardised quality assessment instrument.

### 6.1 Strengths, Limitations and Future Directions

The findings should be interpreted in light of several strengths and limitations. The survey included a large sample of healthcare professionals from an established OMW network across Germany, Austria and Switzerland, combining quantitative and qualitative data to explore current AI use, perceived opportunities, risks and practical applications. The questionnaire was piloted with healthcare professionals and refined accordingly; however, formal assessment of face validity was not performed. Although this may have introduced minor measurement bias, the consistency of responses and convergence between quantitative and qualitative findings suggest that any influence on the overall conclusions was likely limited. Moreover, the cross‐sectional design and reliance on self‐reported practices preclude causal inferences and may not fully reflect practices in other healthcare systems or educational contexts. Furthermore, AI capabilities continue to advance rapidly; therefore, the results reflect clinicians' experiences up to the time of the survey. Future research should evaluate the educational effectiveness of AI‐assisted generation of OMW, identify competencies required for their safe development and review, establish quality assurance and governance frameworks and investigate their impact on workplace learning, knowledge translation and ultimately patient care.

### 6.2 Recommendations for Practice

AI can substantially facilitate the development of workplace‐based educational resources such as OMW. However, AI‐assisted generation of educational materials should undergo peer review before dissemination to verify scientific accuracy, references, visual content and educational appropriateness. Healthcare organisations should strengthen AI competencies among nurses through training in prompt development, critical appraisal of AI‐generated content and structured quality assurance processes.

To promote transparency, complete generation of educational materials by AI should include a brief disclosure and quality statement. For example, ‘This One Minute Wonder was generated using artificial intelligence (ChatGPT, OpenAI). The authors selected the underlying sources, critically reviewed all generated content, and approved the final version. The authors take full responsibility for the accuracy, interpretation, summarisation, and presentation of the underlying evidence. This One Minute Wonder does not replace reading the original publication’. Such disclosures reinforce professional accountability and support trust in AI‐assisted educational resources. When AI is used for specific elements, such as generating figures, content, or layout, these should be reported for each OMW.

## 7 Conclusions

The use of AI for developing educational materials such as One Minute Wonder (OMW) appears to be an established practice among clinicians. AI offers substantial benefits for efficiency, content development and visual design, but it requires rigorous human oversight because inaccuracies, fabricated information and misleading visual content remain common. These results highlight the growing need for AI competencies among nurses and other healthcare professionals—not only to generate AI‐assisted generation of educational materials but also to critically appraise, verify and refine AI‐generated content before its implementation in education and clinical practice.

## Funding

The authors have nothing to report.

## Ethics Statement

According to applicable institutional and national regulations, formal ethics committee approval was not required because the study involved an anonymous survey of healthcare professionals and did not collect sensitive personal data. The study was conducted in accordance with the ethical principles of the Declaration of Helsinki [21].

## Consent

The authors have nothing to report.

## Conflicts of Interest

The authors declare no conflicts of interest.

## Supporting information


**Data S1:** Supporting Information.
**Table S1:** Thematic analysis opportunities.
**Table S2:** Thematic analysis risks and problems.
**Prompt 1:** Visual Abstract Prompt and OMW.
**Prompt 2:** Human‐Centred Narrative Prompt and OMW.
**Prompt 3:** Nursing Values Style Prompt and OMW.

## Data Availability

The data availability statement is available on reasonable request.
