# Supplementary material for: The Use of Artificial Intelligence in Creating Educational Material for Critical Care Clinicians: A Survey
Source: Nurs Crit Care. 2026 Oct 28;31(6):e70753. doi: 10.1111/nicc.70753 (PMC13717210; doi:10.1111/nicc.70753)
Supplement: Supplementary file 1 [file NICC-31-0-s001.docx]

**SUPPLEMENT**

**The Use of Artificial Intelligence in Creating Educational Material for Critical Care**

Nydahl P., Krotsetis, S., Langer G., Krüger L.

**Content**

- Table S1: Thematic analysis opportunities
- Table S2: Thematic analysis risks and problems
- Prompt 1: Visual Abstract Prompt and OMW
- Prompt 2: Human-Centred Narrative Prompt and OMW
- Prompt 3: Nursing Values Style Prompt and OMW
- Q-AI-OMW Checklist
- Survey questionnaire
- CROSS Checklist

**Table S1** Thematic analysis opportunities

| **Main theme** | | |
| --- | --- | --- |
| **AI as a Facilitator of Educational Resource Development and Knowledge Translation** | | |
| **Sub themes** | **Aspects** | **Illustrative content from responses** |
| **1. Efficiency** | Faster creation of OMW | quicker production, reduced workload, faster processing |
|  | Reduced effort in content preparation | rganizatio texts, shortening content, simplifying information |
|  | Efficient updating and dissemination | faster updates, continuous distribution |
| **2. Visualisation** | Image and graphic generation | AI-generated images, infographics, illustrations, tables |
|  | Improved attractiveness and layout | eye-catching designs, modern appearance, branding, corporate design |
|  | Enhanced learning through visual presentation | easier understanding, improved retention, visual learning support |
| **3. Communication** | Text formulation and editing | wording, target-group adaptation, simple language, slogans |
|  | Summarisation and structuring | condensation of complex information, rganization of content |
|  | Translation and multilingual support | translation of studies and content into other languages |
| **4. Knowledge** | Literature search and source identification | finding sources, scientific information retrieval |
|  | Access to current knowledge | rapid access to new knowledge, current standards, evidence summaries |
|  | Information completeness | broader information coverage and topic exploration |
| **5. Creativity** | Idea development and inspiration | brainstorming, generating new perspectives, design ideas |
|  | Innovation and new approaches | opening new fields, creative solutions, alternative presentations |
|  | Support for topic selection and implementation | identifying themes, generating implementation options |

**Table S2: Thematic analysis risks and problems**

| **Main theme: AI as a Potential Threat to the Trustworthiness and Professional Integrity of Educational Resources** | | |
| --- | --- | --- |
| **Sub themes** | **Subthemes** | **Illustrative quotes (British English translation)** |
| **Misinformation** | Incorrect facts; hallucinations; fabricated references; outdated information; non-evidence-based content | “Misinformation, AI-invented data and hallucinations”; “incorrect or outdated data”; “non-evidence-based knowledge transfer”; “factually incorrect statements, missing references” |
| **Need for Verification** | Quality control; validation of outputs; critical appraisal; professional review; overreliance on AI | “Everything must be checked”; “Review is always necessary”; “Control is absolutely essential”; “All content must be reviewed”; “AI must not replace thinking but should support the creation process” |
| **Scientific Quality** | Unclear source provenance; poor-quality sources; insufficient literature appraisal; reduced scientific rigour | “No valid sources; this absolutely must be checked”; “inadvertent use of hallucinated references”; “not always valid”; “verification of the credibility of sources is, of course, necessary”; “that textbooks and professional journals are no longer used” |
| **Loss of Professional Engagement** | Reduced critical thinking; less learning; superficial engagement; neglect of professional skills | “less engagement with the content”; “it encourages people to neglect certain skills with increasing use”; “less independent thinking”; “superficial research – little personal initiative”; “AI must not replace thinking” |
| **Legal and Ethical Concerns** | Data protection; confidentiality; copyright; image rights; transparency of AI use | “data protection issues”; “breaches of data protection”; “copyright infringements”; “problems with image rights”; “no disclosure of AI use and presenting it as one's own work” |
| **Loss of Authenticity** | Reduced individuality; loss of personal style; generic outputs; reduced practical relevance | “the personal touch is missing”; “no individuality”; “individuality is lost”; “little realistic relevance to practice”; “corporate identity is missing” |

**Visual Abstract Prompt and OMW**

**Important Notice**

This prompt is provided as an example of a structured approach for generating educational One-Minute Wonder (OMW) infographics using generative artificial intelligence. The prompt should be adapted to local legal, ethical, regulatory, institutional, and copyright requirements before use.

The generated output is intended exclusively for educational purposes and must undergo expert human review prior to dissemination, publication, or clinical application. Users remain responsible for ensuring scientific accuracy, compliance with applicable laws and regulations, protection of intellectual property rights, and adherence to local governance requirements.

**Standardised Prompt for AI-Assisted Generation of Visual Abstracts for Quantitative Studies**

Create a professional medical visual abstract in A4 portrait format.

Source: the most recently uploaded scientific publication.

The visual abstract should resemble a high-quality scientific visual abstract suitable for publication in leading healthcare journals.

Target audience:
Healthcare professionals, researchers, educators, and decision-makers.

Purpose:
To communicate the key findings of a quantitative study within approximately one minute.

**SCIENTIFIC REPORTING PRINCIPLES**

- Maintain scientific accuracy.
- Use study data exactly as reported.
- Do not invent findings.
- Do not fabricate, modify, or infer numerical values.
- Prioritise primary outcomes over secondary outcomes.
- Report effect sizes exactly as published.
- Report confidence intervals when appropriate.
- Report p-values only if clinically relevant.
- Reflect certainty of evidence when reported.
- Avoid overstating associations as causal relationships.
- Use cautious wording for observational studies.
- Report limitations proportionately.
- Use only bibliographic information explicitly reported in the source publication.

**VISUAL ABSTRACT PRINCIPLES**

- Journal-style visual abstract.
- Inspired by JAMA, NEJM, BMJ, Nature and Critical Care.
- Scientific clarity over artistic expression.
- Minimal decoration.
- Maximum readability.
- Strong visual hierarchy.
- White background.
- High contrast.
- Clean typography.
- Large numerical results.
- Minimal illustrations.
- No unnecessary graphics.

**LAYOUT**

Three-panel structure.

Panel 1:
Study Question

Panel 2:
Key Results

Panel 3:
Clinical Implications

No additional panels.

**PANEL 1 — STUDY QUESTION**

Display:

- Study title (shortened)
- Study design
- Sample size
- Population
- Intervention/exposure
- Comparator (if applicable)

Maximum 30 words.

Visual elements:

- Simple medical icon
- Study design icon
- Population icon

No detailed methods.

**PANEL 2 — KEY RESULTS**

Display only the most important findings.

Prioritise:

1. Primary outcome
2. Main effect size
3. Key secondary outcome

Visual hierarchy:

Largest element:
Primary numerical result

Examples:

OR 0.39

RR 0.74

HR 0.61

−2.1 days

+18%

Include:

- Confidence interval (if available)
- Direction of effect
- Simple visual indicator

Maximum four findings.

No lengthy explanations.

No narrative text.

No speculative interpretation.

**PANEL 3 — CLINICAL IMPLICATIONS**

Summarise implications in no more than three statements.

Each statement:

- Maximum six words
- Action-oriented
- Evidence-aligned

Examples:

- Consider family voice recordings
- Screen for delirium routinely
- Encourage early mobilisation

No recommendations beyond the evidence.

No guideline statements unless explicitly reported.

**TYPOGRAPHY**

- Large title
- Very large primary outcome
- Readable from one metre
- Minimal text
- Maximum 120 words total

**COLOUR USE**

- Limited colour palette
- Professional healthcare appearance
- Use colour only to support interpretation
- Avoid decorative colour schemes

**IMAGES**

- Prefer icons over illustrations.
- Avoid emotional imagery.
- Avoid cartoon-like characters.
- No identifiable persons.
- No copyrighted material.
- No logos.

**TAKE-HOME MESSAGE**

One sentence only.

Maximum 15 words.

Must accurately reflect the primary outcome.

Examples:

Family voice interventions reduced delirium incidence in critically ill adults.

Early mobilisation was associated with improved functional recovery.

**SOURCE**

Authors

Journal

Year

DOI

**DISCLAIMER**

At the bottom:

" This One Minute Wonder was generated using artificial intelligence (ChatGPT, OpenAI). The authors selected the underlying sources, critically reviewed all generated content, and approved the final version. The authors take full responsibility for the accuracy, interpretation, summarisation, and presentation of the underlying evidence. This One Minute Wonder does not replace reading the original publication."

**FINAL QUALITY CHECK**

Verify that:

- All data originate from the publication.
- Numerical values are exact.
- Primary outcomes are prioritised.
- No findings are fabricated.
- Clinical implications remain evidence-based.
- The study can be understood within one minute.
- Source and disclaimer are included.

**OMW 1 Visual Abstract**

**Human-Centred Narrative Prompt and OMW**

**Important Notice**

This prompt is provided as an example of a structured approach for generating educational One-Minute Wonder (OMW) infographics using generative artificial intelligence. The prompt should be adapted to local legal, ethical, regulatory, institutional, and copyright requirements before use.

The generated output is intended exclusively for educational purposes and must undergo expert human review prior to dissemination, publication, or clinical application. Users remain responsible for ensuring scientific accuracy, compliance with applicable laws and regulations, protection of intellectual property rights, and adherence to local governance requirements.

**Standardised Prompt for AI-Assisted Generation of Human-Centred Narrative One-Minute Wonders**

Create a colourful medical One-Minute Wonder infographic in A4 portrait format using a human-centred narrative style.

Use only materials that are legally available for reuse and adaptation under the applicable licence terms.

Source: the most recently uploaded scientific publication.

The infographic should resemble a high-quality educational resource from a university hospital, focusing on patients, families, lived experiences, and person-centred care.

**TARGET AUDIENCE**

Healthcare professionals.

**PURPOSE**

To communicate the human meaning and practical implications of a scientific study within approximately one minute.

The infographic should help healthcare professionals understand:

- What patients experience
- What families experience
- What healthcare professionals can learn
- How care can become more person-centred

**EDUCATIONAL PHILOSOPHY**

Prioritise:

- Human experience
- Personhood
- Meaning
- Reflection
- Empathy
- Compassion
- Humanisation of care

The infographic should encourage reflection and professional learning while remaining scientifically accurate.

**SCIENTIFIC REPORTING PRINCIPLES**

- Maintain scientific accuracy.
- Use study findings exactly as reported.
- Do not invent narratives.
- Do not fabricate quotations.
- Do not exaggerate experiences.
- Reflect uncertainty where appropriate.
- Use only findings supported by the publication.
- Report themes faithfully.
- Preserve the meaning of participants' experiences.
- Avoid causal claims unless supported by the study design.

**HUMAN-CENTRED VALUES**

Where appropriate, reflect:

- Dignity
- Person-centred care
- Compassion
- Respect
- Safety
- Equity
- Inclusion
- Family involvement
- Recovery
- Hope
- Partnership
- Human connection

Visual representations should support diversity and inclusivity.

**DESIGN PRINCIPLES**

- Human-centred rather than data-centred.
- Storytelling through images.
- Warm and welcoming appearance.
- Soft colour palette.
- Emotional resonance.
- Strong visual focus on people.
- Large illustrations.
- Generous white space.
- Maximum 30–40 words per panel.

**STYLE**

- Modern healthcare illustration style.
- Professional and evidence-based.
- Friendly and educational.
- Soft colours.
- Clean lines.
- Diverse and inclusive representations.
- No logos.
- No identifiable real persons.
- No copyrighted characters.
- Avoid stereotypical depictions.

**GENERAL LAYOUT**

Three-panel structure.

Panel 1:
Human Situation

Panel 2:
What Was Learned

Panel 3:
Implications for Care

**PANEL 1 — HUMAN SITUATION**

Focus on:

- Why this topic matters
- Patient or family experience
- Human relevance

Maximum three short statements.

Examples:

- Delirium can be frightening
- Families feel helpless
- Recovery continues after ICU

Visual emphasis:

Large central illustration.

People should be the dominant visual element.

**PANEL 2 — WHAT WAS LEARNED**

Focus on:

- Main themes
- Key insights
- Meaning of findings

Maximum four statements.

Examples:

- Patients remember emotions
- Communication shapes recovery
- Families provide reassurance
- Personhood matters

Use themes rather than statistics when appropriate.

If quantitative results exist, report only the most important result.

**PANEL 3 — IMPLICATIONS FOR CARE**

Focus on actions healthcare professionals can take.

Maximum three statements.

Examples:

- Listen actively
- Include families
- Support recovery
- Explain what is happening
- See the person

Action statements should be short and memorable.

**TEXT RULES**

USE VERY LITTLE TEXT.

- Maximum 120–160 words total.
- No long paragraphs.
- No detailed methods.
- No statistical tables.
- No excessive numerical detail.
- Focus on meaning.
- Focus on practical relevance.
- Prefer simple language.
- Prefer icons and illustrations over text.

**VISUAL PRIORITIES**

Visual hierarchy:

1. People
2. Relationships
3. Key message
4. Supporting information

Illustrations should depict:

- Patients
- Families
- Nurses
- Physicians
- Recovery journeys
- Communication
- Human connection

Avoid excessive technology-focused imagery unless central to the study.

**TAKE-HOME MESSAGE**

One short sentence.

Maximum 15 words.

Should reflect:

- Humanity
- Person-centred care
- Practical learning

Examples:

*A familiar voice can help patients feel safe, connected, and oriented.*

*Former patients are teachers. Their stories improve future care.*

*Seeing the person improves the experience of critical illness.*

**SOURCE**

Authors

Journal

Year

DOI

**DISCLAIMER**

At the bottom:

" This One Minute Wonder was generated using artificial intelligence (ChatGPT, OpenAI). The authors selected the underlying sources, critically reviewed all generated content, and approved the final version. The authors take full responsibility for the accuracy, interpretation, summarisation, and presentation of the underlying evidence. This One Minute Wonder does not replace reading the original publication."

**FINAL QUALITY CHECK**

Verify that:

- All findings originate from the publication.
- No experiences have been invented.
- Human experiences are represented respectfully.
- Person-centred values are preserved.
- The take-home message is supported by the data.
- Diversity and inclusion are respected.
- The infographic can be understood within one minute.
- The source and disclaimer are included.
- The final product supports reflective healthcare learning.

**OMW 2 Narrative style**

**Nursing Values Style Prompt and OMW**

**Important Notice**

This prompt is provided as an example of a structured approach for generating educational One-Minute Wonder (OMW) infographics using generative artificial intelligence. The prompt should be adapted to local legal, ethical, regulatory, institutional, and copyright requirements before use.

The generated output is intended exclusively for educational purposes and must undergo expert human review prior to dissemination, publication, or clinical application. Users remain responsible for ensuring scientific accuracy, compliance with applicable laws and regulations, protection of intellectual property rights, and adherence to local governance requirements.

**Standardised Prompt for AI-Assisted Generation of Nursing Values One-Minute Wonders**

Create a colourful medical One-Minute Wonder infographic in A4 portrait format using a Nursing Values Style.

Use only materials that are legally available for reuse and adaptation under applicable licence terms.

Source: the most recently uploaded scientific publication.

The infographic should resemble a high-quality educational resource from a university hospital and integrate scientific findings with professional nursing values.

**TARGET AUDIENCE**

Healthcare professionals, particularly nurses and other professionals involved in direct patient care.

**PURPOSE**

To communicate scientific findings while highlighting their implications for professional nursing practice, person-centred care, and human dignity.

The infographic should help healthcare professionals understand:

- What the study found
- Why it matters for patients
- What it means for nursing care
- How professional values can be translated into practice

**EDUCATIONAL PHILOSOPHY**

Integrate:

- Evidence-based practice
- Person-centred care
- Clinical judgement
- Professional reflection
- Ethical care

The infographic should connect research findings with everyday nursing actions.

**SCIENTIFIC REPORTING PRINCIPLES**

- Maintain scientific accuracy.
- Use study findings exactly as reported.
- Do not invent findings.
- Do not fabricate quotations.
- Do not exaggerate effects.
- Represent uncertainties appropriately.
- Reflect limitations proportionately.
- Use only information explicitly reported in the publication.
- Avoid causal language unless supported by the study design.
- Prioritise clinically relevant findings.

**CORE NURSING VALUES**

Where appropriate, emphasise:

- Dignity
- Personhood
- Compassion
- Respect
- Safety
- Communication
- Family involvement
- Advocacy
- Trust
- Inclusion
- Equity
- Recovery
- Hope
- Professional accountability

The values should emerge naturally from the findings rather than being artificially imposed.

**DESIGN PRINCIPLES**

- Modern university hospital appearance.
- Warm and professional.
- Evidence-based.
- Human-centred.
- Clinically relevant.
- Easy to understand within one minute.
- Large illustrations.
- Large typography.
- Generous white space.
- Maximum 30–40 words per panel.

**STYLE**

- Contemporary nursing infographic.
- Modern medical illustration style.
- Professional and educational.
- Soft colour palette.
- High visual clarity.
- Diverse and inclusive illustrations.
- No logos.
- No identifiable real persons.
- No copyrighted characters.
- Avoid stereotypes.

**GENERAL LAYOUT**

Three-panel structure.

Panel 1:
Why This Matters

Panel 2:
What We Learned

Panel 3:
What Nurses Can Do

**PANEL 1 — WHY THIS MATTERS**

Focus on:

- Clinical relevance
- Patient perspective
- Human significance

Maximum three statements.

Examples:

- Delirium can be frightening
- Families need information
- Recovery extends beyond discharge

Visual emphasis:

Patient and family experience.

**PANEL 2 — WHAT WE LEARNED**

Present:

- Main findings
- Key themes
- Most important evidence

Maximum four statements.

For quantitative studies:

- Present one key effect size
- Present one key outcome

For qualitative studies:

- Present key themes

Always connect findings to patient experience.

Examples:

- Familiar voices reduce delirium
- Patients value communication
- Recovery takes time
- Personhood matters

**PANEL 3 — WHAT NURSES CAN DO**

Focus on actionable nursing practice.

Maximum three actions.

Examples:

- Explain every procedure
- Include families actively
- Continue communicating
- Support recovery
- Preserve dignity
- See the whole person

Actions should be short, memorable, and evidence-aligned.

**NURSING REFLECTION BOX**

Include a small highlighted box containing:

"Nursing Insight"

Summarise the central professional lesson in one sentence.

Examples:

- Patients remember how we made them feel.
- Communication remains important even when patients cannot respond.
- Small nursing actions may have lasting consequences.

Maximum 15 words.

**TEXT RULES**

USE VERY LITTLE TEXT.

- Maximum 120–160 words total.
- No long paragraphs.
- No detailed methods.
- No excessive statistics.
- Focus on relevance.
- Focus on patient benefit.
- Focus on nursing implications.
- Prefer icons over text.

**VISUAL PRIORITIES**

Visual hierarchy:

1. Patient
2. Nurse
3. Family
4. Key finding
5. Supporting information

Illustrations should emphasise:

- Human connection
- Communication
- Care relationships
- Recovery
- Dignity
- Hope

Technology should remain secondary unless central to the study.

**TAKE-HOME MESSAGE**

One short sentence.

Maximum 15 words.

Examples:

- Seeing the person improves the experience of critical illness.
- Communication is care, even when patients cannot speak.
- Small acts of dignity can have lasting effects.

**SOURCE**

Authors

Journal

Year

DOI

**DISCLAIMER**

At the bottom:

" This One Minute Wonder was generated using artificial intelligence (ChatGPT, OpenAI). The authors selected the underlying sources, critically reviewed all generated content, and approved the final version. The authors take full responsibility for the accuracy, interpretation, summarisation, and presentation of the underlying evidence. This One Minute Wonder does not replace reading the original publication."

**FINAL QUALITY CHECK**

Verify that:

- All findings originate from the publication.
- Nursing implications are evidence-informed.
- Patient dignity is respected.
- Diversity and inclusion are respected.
- No findings are fabricated.
- The take-home message is supported by the data.
- The infographic can be understood within one minute.
- Source and disclaimer are included.
- The final product supports professional nursing practice.

**OMW 3 Nursing Values Style**

**Q-AI-OMW Checklist**

Based on the findings of this study and considerations specific to generative AI, we propose the Q-AI-OMW checklist as a preliminary framework for developing and reviewing AI-assisted OMWs. It addresses five quality domains: educational quality, visual and information design, clinical and scientific quality, AI-specific quality assurance, and responsible and transparent use (supplement). The checklist requires further evaluation and validation before its use as a standardised quality assessment instrument.

**Proposed Quality Checklist for AI-Assisted One-Minute Wonder (Q-AI-OMW)**

| **Domain** | **Criterion** | **Met (Y/N)** |
| --- | --- | --- |
| **1. Educational quality** | |  |
| Clear focus | The OMW addresses one clearly defined topic and communicates one central key message or learning objective. |  |
| Brevity and relevance | Content is concise, relevant, easy to understand, and can be grasped in approximately one minute. |  |
| Comprehensibility and recall | The OMW can be understood without additional explanation and uses appropriate techniques to support rapid recall. |  |
| **2. Visual and information design** | |  |
| Visual clarity | Font sizes, graphics, spacing, and layout support readability and avoid unnecessary visual distraction. |  |
| Logical structure | Headings, text, and visual elements are logically organised and enable rapid orientation. |  |
| **3. Clinical and scientific quality** | |  |
| Clinical relevance | The key message has a clear relevance to clinical practice or the intended target group. |  |
| Scientific accuracy | Statements, numerical data, recommendations, and conclusions accurately reflect the underlying evidence. |  |
| Evidence fidelity | Simplification does not distort the meaning, context, certainty, or limitations of the original evidence, and sources are traceable. |  |
| **4. AI-specific quality assurance** | |  |
| Source fidelity | AI-generated content has been checked against the original source(s) for fabricated, incorrect, misleading, or unsupported information. |  |
| Visual and numerical accuracy | AI-generated or reproduced figures, numbers, illustrations, and diagrams have been checked for accuracy and potential misrepresentation. |  |
| Human verification | All AI-generated content has been critically reviewed and approved by a suitably qualified person before use or publication. |  |
| **5. Responsible and transparent use** | |  |
| Transparency | Authors, date of creation/update, sources, and the use and purpose of generative AI are reported as appropriate. |  |
| Responsible use | Privacy, confidentiality, copyright, intellectual property, bias, and inclusivity have been considered. |  |
| Human responsibility | The human authors retain responsibility for the accuracy, appropriateness, and final content of the OMW. |  |

**Survey questionnaire**

(Translated from German into English using ChatGPT (OpenAI), with the final translation reviewed and approved by the authors)

**OMW and Artificial Intelligence**

**Use of Artificial Intelligence for the Creation of One Minute Wonders**

This anonymous survey within the One Minute Wonder (OMW) Network explores the use of artificial intelligence (AI) for the creation of OMW in healthcare.

The aim is to better understand experiences, opportunities, and challenges associated with AI use.

Estimated completion time: approximately 3–5 minutes.

The data will be analysed statistically. Open-ended responses will be categorised and analysed qualitatively. The findings will be reflected upon and published.

Survey closing date: 21 June 2026.

By participating, you agree to the terms of participation.

Thank you for your support.

<<names of authors>>

**1. Agreement ...**

□ I agree to participate (continue to survey)

□ I do not agree to participate (end survey)

**Demographic Information**

**2. In which area do you mainly work?**

□ Intensive Care Unit (ICU)

□ General Ward

□ Emergency Department

□ Operating Theatre / Anaesthesia

□ Diagnostic or Treatment Services

□ Education / Teaching

□ Management

□ Other

□ Prefer not to answer

**3. Which professional group do you belong to?**

□ Nursing

□ Medicine

□ Other

□ Prefer not to answer

**4. What is your highest educational qualification?**

□ Vocational qualification / professional training

□ Academic degree

□ Prefer not to answer

**Use of AI for OMW Development**

**5. Have you ever used AI to create OMW?**

□ Yes

□ No

(Note to readers and not displayed on survey: If participants selected “Yes”, they continue with next question 6, if they selected “No”, they continue with question 23)

**6. How many OMW have you created using AI?**

(Please enter a whole number; enter 0 if applicable.)

**7. For what purposes do you use AI when creating OMW?**

(Multiple answers possible)

□ Text generation

□ Summarising scientific studies

□ Translation

□ Image generation

□ Graphics / infographics

□ Layout ideas

□ Formulating key learning messages

□ Literature searching

□ Other (please specify)

**8. Which AI systems do you use when creating OMW?**

(Multiple answers possible)

□ ChatGPT

□ Microsoft Copilot

□ Gemini

□ Claude

□ Canva AI

□ Midjourney

□ DALL·E

□ Other (please specify)

**9. Which versions do you use?**

(Multiple answers possible)

□ I use only free versions

□ I use paid (premium) versions

□ I use both free and paid versions depending on the AI system

□ Other (please specify)

□ None of the above

**10. How frequently do you use AI when creating OMW?**

(Please interpret the categories as proportions, regardless of the total number of OMW you have created.)

□ Rarely (approximately 0–1 out of 10 OMW)

□ Occasionally (approximately 2–3 out of 10 OMW)

□ Regularly (approximately 4–6 out of 10 OMW)

□ Frequently (approximately 7–8 out of 10 OMW)

□ Almost always (approximately 9–10 out of 10 OMW)

**Use of Scientific Sources**

**11. When creating OMW, how often do you upload scientific publications (text copies or PDFs) to AI systems?**

□ Rarely (approximately 0–1 out of 10 OMW)

□ Occasionally (approximately 2–3 out of 10 OMW)

□ Regularly (approximately 4–6 out of 10 OMW)

□ Frequently (approximately 7–8 out of 10 OMW)

□ Almost always (approximately 9–10 out of 10 OMW)

**12. What types of content do you upload?**

(Multiple answers possible)

□ Scientific articles

□ Abstracts

□ Clinical guidelines

□ Presentations

□ My own texts

□ Images / graphics

□ Anonymised case data

□ No uploads

□ Other (please specify)

**13. How often do you consider data protection or copyright issues when using AI?**

□ Rarely (approximately 0–1 out of 10 OMW)

□ Occasionally (approximately 2–3 out of 10 OMW)

□ Regularly (approximately 4–6 out of 10 OMW)

□ Frequently (approximately 7–8 out of 10 OMW)

□ Almost always (approximately 9–10 out of 10 OMW)

**Quality Assurance**

**14. How often do you critically review AI-generated content in OMW that you create yourself?**

□ Rarely

□ Occasionally

□ Regularly

□ Frequently

□ Almost always

**15. How often do you identify factual errors or inaccuracies during the AI-assisted creation of OMW?**

□ Rarely

□ Occasionally

□ Regularly

□ Frequently

□ Almost always

**16. Which types of errors occur most frequently?**

(Multiple answers possible)

□ Incorrect content

□ Fabricated references

□ Incorrect numerical data

□ Ambiguous wording

□ Inappropriate images

□ Outdated information

□ Oversimplification

□ No problems identified

□ Other (please specify)

**17. How often do AI-generated OMW require revision?**

□ Rarely

□ Occasionally

□ Regularly

□ Frequently

□ Almost always

**Perceptions and Evaluation**

**18. AI improves the creation of OMW.**

□ Strongly agree

□ Agree

□ Neither agree nor disagree

□ Disagree

□ Strongly disagree

**19. AI saves time when creating OMW.**

□ Strongly agree

□ Agree

□ Neither agree nor disagree

□ Disagree

□ Strongly disagree

**20. AI-generated content carries risks of misinformation.**

□ Strongly agree

□ Agree

□ Neither agree nor disagree

□ Disagree

□ Strongly disagree

**21. Should AI-generated content be labelled as such?**

□ Yes

□ No

□ Unsure

**22. How would you rate your own competence in using AI?**

□ Very low

□ Low

□ Moderate

□ High

□ Very high

**Future Perspectives and Open Questions**

**23. What opportunities do you see for AI in the creation of OMW?**

**24. What risks or problems do you perceive?**

**25. What support would you like to receive?**

(Multiple answers possible)

□ Training courses

□ Guidelines

□ Quality assurance checklists

□ Data protection recommendations

□ Example prompts

□ Image databases

□ Exchange within the network

□ No support needed

□ Other (please specify)

**Final Questions**

**26. Would you use AI-generated OMW in routine clinical practice?**

□ Yes

□ No

□ Unsure

**27. Have you ever encountered the dissemination of incorrect AI-generated content?**

□ Yes

□ No

□ Unsure

If yes, please describe:

Thank you for participating.

The results will help improve understanding of the opportunities and risks associated with AI use within the OMW Network and support the development of future quality standards.

**Checklist for Reporting Of Survey Studies (CROSS)**

| **Section/topic** | **Item** | **Item description** | **Reported on page #** |
| --- | --- | --- | --- |
| **Title and abstract** | | |  |
| Title and abstract | 1a | State the word “survey” along with a commonly used term in title or abstract to introduce the study’s design. | 1 |
|  | 1b | Provide an informative summary in the abstract, covering background, objectives, methods, findings/results, interpretation/discussion, and conclusions. | 1 |
| **Introduction** | | |  |
| Background | 2 | Provide a background about the rationale of study, what has been previously done, and why this survey is needed. | 3 |
| Purpose/aim | 3 | Identify specific purposes, aims, goals, or objectives of the study. | 4 |
| **Methods** | | |  |
| Study design | 4 | Specify the study design in the methods section with a commonly used term (e.g., cross-sectional or longitudinal). | 4 |
|  | 5a | Describe the questionnaire (e.g., number of sections, number of questions, number and names of instruments used). | 4-5 |
| Data collection methods | 5b | Describe all questionnaire instruments that were used in the survey to measure particular concepts. Report target population, reported validity and reliability information, scoring/classification procedure, and reference links (if any). | 5 |
|  | 5c | Provide information on pretesting of the questionnaire, if performed (in the article or in an online supplement). Report the method of pretesting, number of times questionnaire was pre-tested, number and demographics of participants used for pretesting, and the level of similarity of demographics between pre-testing participants and sample population. | 5 |
|  | 5d | Questionnaire if possible, should be fully provided (in the article, or as appendices or as an online supplement). | Suppl |
| Sample characteristics | 6a | Describe the study population (i.e., background, locations, eligibility criteria for participant inclusion in survey, exclusion criteria). | 5 |
|  | 6b | Describe the sampling techniques used (e.g., single stage or multistage sampling, simple random sampling, stratified sampling, cluster sampling, convenience sampling). Specify the locations of sample participants whenever clustered sampling was applied. | 5 |
|  | 6c | Provide information on sample size, along with details of sample size calculation. | 5 |
|  | 6d | Describe how representative the sample is of the study population (or target population if possible), particularly for population-based surveys. | n/a |
| Survey  administration | 7a | Provide information on modes of questionnaire administration, including the type and number of contacts, the location where the survey was conducted (e.g., outpatient room or by use of online tools, such as SurveyMonkey). | 5 |
|  | 7b | Provide information of survey’s time frame, such as periods of recruitment, exposure, and follow-up days. | 5 |
|  | 7c | Provide information on the entry process:  –>For non-web-based surveys, provide approaches to minimize human error in data entry.  –>For web-based surveys, provide approaches to prevent “multiple participation” of participants. | 5 |
| Study preparation | 8 | Describe any preparation process before conducting the survey (e.g., interviewers’ training process, advertising the survey). | 5-6 |
| Ethical considerations | 9a | Provide information on ethical approval for the survey if obtained, including informed consent, institutional review board [IRB] approval, Helsinki declaration, and good clinical practice [GCP] declaration (as appropriate). | 5-6 |
|  | 9b | Provide information about survey anonymity and confidentiality and describe what mechanisms were used to protect unauthorized access. | 5 |
| Statistical  analysis | 10a | Describe statistical methods and analytical approach. Report the statistical software that was used for data analysis. | 5 |
|  | 10b | Report any modification of variables used in the analysis, along with reference (if available). | n/a |
|  | 10c | Report details about how missing data was handled. Include rate of missing items, missing data mechanism (i.e., missing completely at random [MCAR], missing at random [MAR] or missing not at random [MNAR]) and methods used to deal with missing data (e.g., multiple imputation). | 5 |
|  | 10d | State how non-response error was addressed. | 5 |
|  | 10e | For longitudinal surveys, state how loss to follow-up was addressed. | n/a |
|  | 10f | Indicate whether any methods such as weighting of items or propensity scores have been used to adjust for non-representativeness of the sample. | n/a |
|  | 10g | Describe any sensitivity analysis conducted. | 5 |
| **Results** | | |  |
| Respondent characteristics | 11a | Report numbers of individuals at each stage of the study. Consider using a flow diagram, if possible. | 6 |
|  | 11b | Provide reasons for non-participation at each stage, if possible. | 6 |
|  | 11c | Report response rate, present the definition of response rate or the formula used to calculate response rate. | 6 |
|  | 11d | Provide information to define how unique visitors are determined. Report number of unique visitors along with relevant proportions (e.g., view proportion, participation proportion, completion proportion). | 6 |
| Descriptive  results | 12 | Provide characteristics of study participants, as well as information on potential confounders and assessed outcomes. | 6 |
| Main findings | 13a | Give unadjusted estimates and, if applicable, confounder-adjusted estimates along with 95% confidence intervals and p-values. | n/a |
|  | 13b | For multivariable analysis, provide information on the model building process, model fit statistics, and model assumptions (as appropriate). | n/a |
|  | 13c | Provide details about any sensitivity analysis performed. If there are considerable amount of missing data, report sensitivity analyses comparing the results of complete cases with that of the imputed dataset (if possible). | 7 |
| **Discussion** | | |  |
| Limitations | 14 | Discuss the limitations of the study, considering sources of potential biases and imprecisions, such as non-representativeness of sample, study design, important uncontrolled confounders. | 11-12 |
| Interpretations | 15 | Give a cautious overall interpretation of results, based on potential biases and imprecisions and suggest areas for future research. | 12-13 |
| Generalizability | 16 | Discuss the external validity of the results. | 10-11 |
| **Other sections** | | |  |
| Role of funding source | 17 | State whether any funding organization has had any roles in the survey’s design, implementation, and analysis. | 13 |
| Conflict of interest | 18 | Declare any potential conflict of interest. | 13 |
| Acknowledgements | 19 | Provide names of organizations/persons that are acknowledged along with their contribution to the research. | 13 |

Sharma A, Minh Duc NT, Luu Lam Thang T, Nam NH, Ng SJ, Abbas KS, Huy NT, Marušić A, Paul CL, Kwok J, Karbwang J, de Waure C, Drummond FJ, Kizawa Y, Taal E, Vermeulen J, Lee GHM, Gyedu A, To KG, Verra ML, Jacqz-Aigrain ÉM, Leclercq WKG, Salminen ST, Sherbourne CD, Mintzes B, Lozano S, Tran US, Matsui M, Karamouzian M. A Consensus-Based Checklist for Reporting of Survey Studies (CROSS). J Gen Intern Med. 2021 Oct;36(10):3179-3187. doi: 10.1007/s11606-021-06737-1. Epub 2021 Apr 22.

<https://pubmed.ncbi.nlm.nih.gov/33886027/>
